# Conceptualising the technical relationship of animal disease surveillance to intervention and mitigation as a basis for economic analysis

**DOI:** 10.1186/1472-6963-11-225

**Published:** 2011-09-19

**Authors:** Barbara Häsler, Keith S Howe, Katharina DC Stärk

**Affiliations:** 1 Veterinary Clinical Sciences, Royal Veterinary College, Hawkshead Lane, North Mymms, Hatfield AL9 7TA, UK; 2 Centre for Rural Policy Research, College of Social Sciences and International Studies, University of Exeter, Devon, EX4 4QE, UK

## Abstract

**Background:**

Surveillance and intervention are resource-using activities of strategies to mitigate the unwanted effects of disease. Resources are scarce, and allocating them to disease mitigation instead of other uses necessarily involves the loss of alternative sources of benefit to people. For society to obtain the maximum benefits from using resources, the gains from disease mitigation must be compared to the resource costs, guiding decisions made with the objective of achieving the optimal net outcome.

**Discussion:**

Economics provides criteria to guide decisions aimed at optimising the net benefits from the use of scarce resources. Assessing the benefits of disease mitigation is no exception. However, the technical complexity of mitigation means that economic evaluation is not straightforward because of the technical relationship of surveillance to intervention. We argue that analysis of the magnitudes and distribution of benefits and costs for any given strategy, and hence the outcome in net terms, requires that mitigation is considered in three conceptually distinct stages. In Stage I, 'sustainment', the mitigation objective is to sustain a free or acceptable status by preventing an increase of a pathogen or eliminating it when it occurs. The role of surveillance is to document that the pathogen remains below a defined threshold, giving early warning of an increase in incidence or other significant changes in risk, and enabling early response. If a pathogen is not contained, the situation needs to be assessed as Stage II, 'investigation'. Here, surveillance obtains critical epidemiological information to decide on the appropriate intervention strategy to reduce or eradicate a disease in Stage III, 'implementation'. Stage III surveillance informs the choice, timing, and scale of interventions and documents the progress of interventions directed at prevalence reduction in the population.

**Summary:**

This article originates from a research project to develop a conceptual framework and practical tool for the economic evaluation of surveillance. Exploring the technical relationship between mitigation as a source of economic value and surveillance and intervention as sources of economic cost is crucial. A framework linking the key technical relationships is proposed. Three conceptually distinct stages of mitigation are identified. Avian influenza, salmonella, and foot and mouth disease are presented to illustrate the framework.

## Background

The broad use of animal disease surveillance (definitions of key terms used can be found in Appendix 1) is illustrated by numerous systems in place worldwide. Surveillance is used for early warning when disease (re-)occurs, to detect infection or disease, to measure prevalence or incidence of pathogens or hazards found in animal populations or along the food chain, to inform intervention activities to reduce or eradicate disease, and to document freedom from disease, infection or the level of chemical contaminants in food products. In a broader sense, surveillance can be considered as a scientific, factual tool that informs policy decisions and the allocation of resources for disease control [1].

This paper originates from a research project that aimed to develop a conceptual framework and practical tool for the economic evaluation of surveillance. The development of a generic economic framework independent of the pathogen, animal species, and surveillance approach or design, demands understanding of the technical relationship between the components of mitigation that impact on the economic value of surveillance. The aim was to explore the relationship between surveillance, intervention and mitigation systematically as a foundation for empirical research into the economic value of mitigation.

In economic terms, animal production systems exist to provide economic value, i.e. the sense of personal well-being or benefit gained by people as a result of consuming animal goods or services created by the transformation of resources. People not only derive substantial value from animal products such as eggs, meat, wool, or leather, but also from animals kept as pets, used for sports, work, or research. Disease reduces the quantity of outputs produced from the resources committed to animal production and thus the benefits people obtain from them. As a result, additional resources are needed to mitigate such negative effects. Mitigation, sometimes regarded as synonymous with control, is defined as the process of making the effects of disease less severe by avoiding, containing, reducing or removing it. Both surveillance and intervention are resource-using activities that are part of a mitigation strategy. Effective surveillance helps to offset negative effects of disease on animal and food production by promoting successful interventions. In assessing the rationality of any resource-using decision, the key criterion is whether the value of outputs consequently recovered is at least sufficient to cover the additional resource costs. Thus the cost of resources committed to mitigation should at least be compensated by the value of the resulting recovered outputs and, ideally, the net benefits (total output value recovered minus total mitigation costs) should be maximised, thus optimising economic efficiency.

The scale and combinations in which surveillance and intervention resources are used determine the total costs and benefits of mitigation, and thus the net effect on people's economic well-being. Technical efficiency refers to the physical relation between resources used and the related outcome and is a prerequisite for economic efficiency [2]. Therefore, the precursor to economic appraisal is acquired understanding of the technical relationships between surveillance, intervention and mitigation.

There is a wide range of definitions, concepts and characteristics of surveillance available. Moreover, several classification systems for surveillance are in place that focus on surveillance approach, design, management, networking and epidemiological criteria [3-5]. Even though such systems are useful in understanding the approach, structure and design of surveillance systems, they do not systematically address the technical relationship between key elements of mitigation essential to the economic analysis of mitigation. To the authors' knowledge, no economic study has explicitly considered the basic technical relationship between surveillance, intervention and mitigation; aggregate conceptual units which are the fundamental elements for consideration when making recommendations for disease mitigation policies which are economically efficient.

Surveillance provides information to guide decisions about the nature and scope of interventions aimed at prevalence or incidence reduction. In general, any information system is designed for problem solving in a social system. The data collection and analysis that contribute to the provision of information to policy makers should always be built on a solid conceptual base [6]. Factors including the frequency and methods of data collection, and the related levels of personnel and institutional infrastructure needed, depend on the quality and scope of information required by policy makers to support their decision-making.

Because the economic assessment of animal disease surveillance must explicitly acknowledge the relationship between disease mitigation, a process that enhances economic benefits, and mitigation resources, a source of economic costs, it also provides essential guidance for the design of suitable information systems.

Therefore, the objective of this article is to show how the technical relationships between animal disease surveillance, intervention and mitigation can be integrated as a conceptual framework to guide economic analysis in practical applications. The conceptual framework presented builds on logical reasoning, practical experience and observations of how mitigation processes evolve over time in animal health services.

## Discussion

### Technical relationship between animal disease surveillance, intervention and mitigation

**Figure 1:**
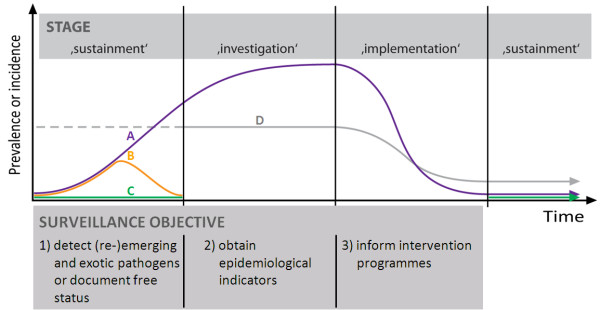
**Schematic illustration of surveillance objectives in relation to a three stage mitigation system**. A: (re-)emerging or exotic epidemic pathogen that is not controlled by response measures, B: (re-)emerging or exotic epidemic pathogen that is controlled by response measures, C: continuous free status, D: endemic disease, dotted line: true value unknown.

The mitigation process can be divided into three stages: sustainment, investigation and implementation (Figure 1). Each stage is defined by specific mitigation objectives and has implications for the pursuit of economic efficiency.

The economic objective of mitigation is independent of the pathogen, animals and animal-derived products, or surveillance approach or design. At any stage, the principal surveillance objective is to provide information to policy makers to support intervention decisions. Importantly, the three stages are a continuum, the starting point for analysis found anywhere on this mitigation continuum depending on the pathogen in question and the specific disease situation.

Targeted pathogens include infectious diseases in animals, zoonotic diseases, food-borne hazards, vector-borne infections, and resistant pathogens and resistance genes. Their categorisation into endemic, (re-)emerging or exotic depends on the initial disease status of a country. Certain endemic diseases may have been present for a long time, while others may have emerged and become endemic, because there were no or insufficient mitigation measures in place. For the purposes of this paper, the starting point is set in the sustainment stage, an actual situation for many diseases in developed countries.

At the start of the mitigation cycle, a pathogen is viewed either as not present in the unit of interest (e.g. farm, region, country) or present at an acceptable level. In the 'sustainment' stage, the mitigation objective is to keep the free or acceptable status either by preventing an increase in incidence of a pathogen or by eliminating a pathogen quickly when it occurs. The surveillance function is therefore to document that a pathogen remains below a defined threshold, and to provide early warning of an increase in incidence or other significant changes in risk (e.g. higher pathogenicity, new subtype). Early warning may trigger a rapid response to contain an increase in incidence of the pathogen (e.g. disease outbreak). If the response measures are insufficient to contain the pathogen, a change in strategy is needed. Mitigation activities switch to the 'investigation' stage. The objective of this second stage is to assess the situation as a forerunner to providing guidance for intervention activities in the subsequent 'implementation' stage. 'Investigation' stage surveillance is therefore to obtain critical epidemiological information, for example about disease incidence or prevalence and the direction and rate of dispersion. Such information is to inform decisions about the intervention strategy appropriate to reduce or eradicate a pathogen. At the 'implementation' stage, the objective is to reduce the prevalence of a pathogen in relation to a defined target based on epidemiologic, economic or political criteria by implementing intervention measures. The corresponding surveillance is then to inform the choice, timing and scale of intervention measures and to document the progress in the light of interventions. Finally, after successful intervention the mitigation objective may again revert to the sustained absence of disease.

In the following sections, the three mitigation stages, related surveillance and intervention as well as the transitions between the stages are described in more detail.

#### Surveillance in Stage I mitigation ('sustainment')

##### A. Mitigation objective

In Stage I, the level of risk must be perceived to be acceptable by decision-makers. The ideal risk would be zero, but in its absence an acceptable level of risk is generally defined as a situation for which no special intervention activities need to be directed at the pathogen [7]. The acceptable status may be a historically free status, freedom from disease, freedom from infection, or contamination of food products maintained below a defined threshold. Policy makers also may be aware of certain endemic pathogens, but categorise them as low priority and therefore do not tackle them. In short, the Stage I mitigation objective is to sustain the acceptable status. Additionally, compliance with international regulations to document disease freedom may facilitate access to foreign markets.

##### B. Surveillance and intervention

Surveillance information is used to document that a pathogen is not present or only in less than a specified proportion of the population, that an endemic status remains stable and to give an early warning signal if the situation is altered. Even though intervention measures are generally not needed in this stage, they are anticipated to combat a pathogen quickly when it occurs and are usually laid down in national contingency plans or equivalent regulations. If surveillance gives an alarm, response interventions will be implemented to contain the disease and prevent further spread (e.g. outbreak control). In such cases, the free status may be (temporarily) suspended until response activities effectively contain the disease. Classical intervention measures for infectious diseases include testing-and-culling, movement bans, quarantine, and emergency vaccination.

According to the World Organisation for Animal Health (OIE), a country, region or zone can declare itself historically or officially free from disease or infection provided it presents evidence based on surveillance information [8]. European Union (EU) regulations stipulate specific requirements for their member countries to document disease freedom using surveillance. Owing to the continuing costs of such surveys, these requirements have triggered efforts to demonstrate disease freedom using novel, more efficient designs such as risk-based sample size calculation of consecutive national surveys [9] or the integration of multiple sources of random and non-random surveillance data in stochastic scenario tree models [10]. In food safety, surveillance is an established tool to demonstrate that chemical contaminants (e.g. pesticides, veterinary drugs, food additives) do not exceed critical values in food products [11].

The surveillance approach and design chosen may vary over time. Changes in external factors such as the international disease situation (e.g. increase in geographical distribution and worldwide incidence of a specific disease) or environmental or behavioural patterns that facilitate the introduction and spread of a pathogen (e.g. establishment of new insect vectors due to climate change, conversion of rain forests into farmland) as well as political priorities and trends may impact on real and perceived risks. Hence there may be a shift from a situation with minimal risk of pathogen incursion or augmentation, and consequently a low level of alert to one where higher vigilance is required. Many surveillance designs, such as sentinel, risk-based or syndromic surveillance have the ability to detect rare cases and can be highly sensitive.

Some endemic diseases relevant to public health are notifiable but not subject to systematic surveillance. In such cases there are sporadic surveillance data about cases occurring in the population, but the true prevalence is generally not known. For example, toxoplasmosis in animals is notifiable in Switzerland, but not subject to systematic surveillance.

##### C. Transition

Should Stage I surveillance and response measures fail, adaptation of mitigation to contain an epidemic will be required and the next two stages may have to be considered. The transition in that case is not clear-cut and depends on various factors, the most important one likely being the ability of decision-makers to assess the situation and promote a change in strategy. No specific surveillance information will be available for endemic pathogens not of high priority in stage I. However, endemic pathogens may move up the priority list due to changes in the international disease situation (e.g. neighbouring countries successfully implementing intervention programmes), an increase in knowledge about the pathogens, the eradication of other public health pathogens and/or the availability of new technologies. Another reason for a shift in priorities may be political preferences and the availability of resources. Any change in priority will cause a transition to Stage II.

#### Surveillance in Stage II mitigation ('investigation')

##### A. Mitigation objective

In Stage II, problem analysis is needed to understand the problem and guide decision-making as a precursor to Stage III mitigation for both endemic and epidemic pathogens. In the process, alternative strategies are assessed taking into account technical, social, economic, institutional and/or management considerations. Finally, a decision is made about whether to implement Stage III mitigation or not.

##### B. Surveillance and intervention

Stage II surveillance is used to obtain epidemiological indicators such as prevalence, incidence, morbidity, mortality, geographical distribution, and frequency of risk or preventive factors. The data provided are a quantitative basis to help policy makers decide if intervention measures are needed and to inform the selection of the appropriate intervention strategy to reduce prevalence of disease. It describes the initial conditions and serves as the foundation for future intervention. Response measures from Stage I may be continued, while further surveillance data are collected to inform alternative or complementary strategies. Sometimes, intervention measures may be pilot-tested to assess the effectiveness of putative interventions.

National or international surveys are sources for baseline and comparable values for prevalence or incidence of pathogens found in animal populations or along the food chain. Moreover, they are used to assess the geographical distribution and to quantify risk or preventive factors or other epidemiological indicators. Depending on both the pathogen and national characteristics, such as the professional standard of the animal health service and availability of animal databases and resources, the sampling design may be probabilistic or non-probabilistic. Random or probability sampling implies choosing the sampling unit (e.g. individuals, herds, farms, slaughterhouses, administrative areas) such that each has the same chance of selection [12]. A range of sampling methods is available, such as simple random, systematic, stratified, cluster or multi-stage sampling. In the non-probability design, the sample is usually selected on the basis of the accessibility or the purposive judgment of the researcher.

In laboratory-based surveillance, collaboration among national and international laboratories enables sharing of various types of epidemiological and pathogen-specific information to produce high quality data. Serotyping or molecular subtyping of pathogens provides important epidemiological data about the infectious agent [13]. This is valuable information contributing to the investigation of the source and risk of infection and the design of effective intervention strategies.

##### C. Transition

The surveillance information feeds into technical, social, economic, institutional and/or management considerations that impact on the decision to implement an intervention programme and thus the transition to Stage III. If Stage II surveillance demonstrates no immediate need to act, decision makers may decide to wait and gather further data to inform decisions about the specific nature of future intervention programmes. If there is insufficient knowledge about a pathogen and/or the technical or financial resources necessary to acquire it are unavailable, surveillance information will contribute to the general body of knowledge, increasing disease awareness and laboratory expertise, but there will not yet be a transition to Stage III. Otherwise, if Stage III mitigation is shown to be feasible and beneficial, the decision is made to move on to that next stage.

#### Surveillance in Stage III mitigation ('implementation')

##### A. Mitigation objective

In Stage III mitigation, the focus is on problem resolution, where the planned intervention strategies are implemented to reduce or eradicate a pathogen. The strategy and targets are well-defined, and the elements necessary to support the mitigation process such as finances, infrastructure, expertise, information networks, and data flow have been taken into account. Further, surveillance and intervention activities have been clearly defined.

##### B. Surveillance and intervention

Stage III surveillance provides essential input for programmes established to reduce or eradicate diseases and to enhance food safety. It is an essential tool throughout the whole stage and its objective changes over time. First, it is used to identify animals or herds eligible for intervention. Surveillance data can classify animals or holdings as infected or non-infected and thus mark them as intervention subjects. Second, surveillance is also used to monitor the progress and effectiveness of intervention measures (mid-term evaluation) and, ultimately, to verify their success (final evaluation). For example, it can be used to check the proportion of immunised animals after a vaccination campaign, or to antigen test newborn animals expected to be free from infection. There is a wide range of intervention measures available to reduce or eradicate a pathogen. These include culling or medical treatment of diseased animals, vaccination, vector control, promotion of resistant breeds, and deliberate exposure to infected animals to promote natural immunisation. They are often flanked by information and awareness-raising campaigns, on-farm bio-security and reorganisation of structures that impact on disease spread, such as live animal markets or transportation systems. If surveillance data suggest that the change in prevalence is not as large as expected, the necessary steps can be taken to implement corrective measures.

The surveillance design needs to be flexible over time depending on the pathogen, mitigation target, and progress of the intervention programme. Thus, the sampling scheme, sample size, and selection of diagnostic tests may change with decreasing prevalence. The OIE grants official recognition of freedom from bovine spongiform encephalopathy, foot and mouth disease (FMD), rinderpest and contagious bovine pleuropneumonia [8] and stipulates detailed surveillance requirements to provide evidence for disease freedom. For all other diseases, it is the responsibility of individual countries to select appropriate surveillance strategies to demonstrate that an intervention has been successful.

##### C. Transition

The attainment of the mitigation target defines the endpoint of Stage III. However, after many years of Stage III mitigation activities the programme may become institutionalised and stagnate in Stage III instead of moving to Stage I. Surveillance that keeps the effects of intervention under review will provide information to policy makers to support the decision about the right time to cease the programme. If decision-makers opt for the transition to Stage I, mitigation activities will focus once again on sustaining an acceptable level of a pathogen.

#### The cycle is complete

The favourable free status may be kept for a prolonged time period and possibly indefinitely. However, all pathogens that are not present or exist at a very low level because of historical freedom or successful mitigation have the potential to (re-)occur, spread and become endemic if mitigation measures are not adequate or sufficient. Thus it is essential to keep mitigation and its related surveillance activities flexible, up-to-date, and equipped to respond adequately to dynamic challenges.

### Three examples to illustrate the conceptual framework

#### 1. Avian influenza

With the emergence of highly pathogenic avian influenza (HPAI) H5N1 in South-East Asia in the past decade and its spread to Europe, policy makers recognised the need for multidisciplinary surveillance teams that detect the virus early to limit its spread, clinical effects, and economic costs. The Food and Agricultural Organisation of the United Nations implemented an early warning surveillance system for the worldwide integration and exchange of avian influenza information [14]. The EU quickly introduced new legislation to accommodate the altered risk. Many countries that have never had a case of HPAI implemented extensive surveillance system in wild birds and poultry to detect an incursion of HPAI quickly and to be able to contain an outbreak without delay. The strategy proved to be successful, as all sporadic HPAI outbreaks in EU member states could be contained within a few months using classical response measures, i.e. all countries stayed at Stage I. However, in other regions of the world, similar measures were unsuccessful. For example in China, Vietnam, Egypt and Indonesia, the disease spread widely despite the implementation of response measures [15]. The situation in Vietnam where HPAI was reported for the first time in 2003 illustrates the consequences of Stage I failure.

##### A. Mitigation objective

The current objective of the Vietnamese programme against HPAI H5N1 is the 'sustained country-wide elimination of the virus' [16].

##### B. Surveillance and intervention

After detecting the disease for the first time in 2003, Vietnam implemented a stamping out programme for infected and at-risk flocks to control the disease [15]. However, culling 45 million poultry failed to eliminate infection and prevent human cases, so it was necessary to adapt the strategy. Surveillance information was gathered to inform the development of a vaccination campaign that aimed at complementing existing measures [16]. Vietnam's integrated national operational programme for avian influenza from 2006 to 2010 outlined an intervention programme with four pillars: 1) rapid identification and response to disease outbreaks, 2) risk-based vaccination, 3) enhanced management, and 4) control of poultry movements and development of disease-free compartments [17]. Surveillance activities for HPAI in Vietnam foresaw clinical case reporting, surveys on markets and slaughterhouses to improve knowledge of virus circulation, and mapping of temporal and spatial distributions of wild birds [17]. Further, surveillance was conducted to assess vaccination protection and to investigate the cause and implement corrective measures if the results were not satisfactory [16]. It was also used to demonstrate whether viruses were still circulating and to assess their antigenic makeup and their distribution. Based on new information that is continuously becoming available, Vietnamese animal health authorities have been modifying the intervention programme to increase its effectiveness.

##### C. Transition

Vietnam may cease vaccination once the risk of infection has significantly decreased, if surveillance and disease reporting systems manage to detect and investigate all cases of suspected HPAI, and if production and marketing methods that are risk factors for virus transmission have been changed [16].

##### Summary

Vietnam is currently in Stage III mitigation aiming at eradicating HPAI. After successful eradication, it is expected to move from Stage III to Stage I mitigation and related surveillance.

#### 2. Salmonella in the European Union

EU regulation 2160/2003 laid the foundation for enhanced food safety by obliging member states to run national control programmes to reduce salmonella in poultry and pigs. Its purpose was to 'ensure that proper and effective measures are taken to detect and control salmonella'. It provided a framework for the definition of targets, the approval of mitigation programmes, and the adoption of rules regarding intervention methods and trade. For each target group (breeding flocks, laying hens, broilers, turkeys, breeding and fattening pigs) it was envisaged to conduct a baseline survey, define reduction targets for all member states and to implement national mitigation programmes to reduce prevalence in the EU. The mitigation programme in laying hens illustrates Stage II and III activities.

##### A. Mitigation objective

The two main objectives of the programme were to set EU targets for the reduction of salmonella and to achieve the defined targets by implementing national mitigation programmes.

##### B. Surveillance and intervention

The primary objective of the baseline survey in laying hens was to estimate the prevalence of *Salmonella *spp. in commercial large-scale holdings to inform setting EU targets. Other objectives were to investigate the relative sensitivity of faecal and environmental samples, the role of vaccination and to collect additional epidemiological information such as serotypes and flock sizes [18]. The EU decision 2004/665 laid down requirements regarding the sampling frame, laboratory analysis, data collection, analysis and communication. Results from the baseline survey were used to stipulate mitigation targets (Regulation 1168/2006) and all EU member states were obliged to submit plans for their national programmes setting out the intervention measures envisaged. After getting the approval from the European Commission, member states implemented surveillance to detect *Salmonella *spp. and the related interventions following case detection. The EU regulation 1168/2006 outlines the surveillance scheme necessary to 'verify the achievement of the Community target for the reduction of salmonella'. Thus, surveillance data provided during this phase are not only an important element for effective and successful intervention, but are also used to check the progress of the intervention programmes.

##### C. Transition

Results from the baseline survey were used to define EU targets and to design national mitigation plans for the reduction of salmonella. Member states implemented these plans and consequently have moved on to Stage III. Once the targets for *Salmonella *spp. in laying hens are achieved, transition to Stage I may be considered and the sampling protocols adapted accordingly.

##### Summary

While the EU mitigation programme for salmonella in laying hens and related surveillance and intervention measures have passed from Stage II to Stage III, the situation is different for pigs where decisions regarding the setting of control targets and the implementation of intervention strategies are under discussion.

#### 3. Foot and mouth disease in Europe

Foot and mouth disease was endemic in Europe from the 17^th ^until the mid 20^th ^century. The development of effective vaccines allowed implementation of vaccination campaigns that reduced the number of FMD outbreaks from almost 900,000 in 1951/52 to 34 between 1977 and 1987 [19]. These campaigns were accompanied by on-farm surveillance, import restrictions and outbreak response measures. In the late 1980s, vaccination was forbidden in Denmark, the UK and Ireland, while the other nine EU member states were still using vaccination. At the same time, evidence accumulated that there were no endemic foci in the EU member states anymore [20]. Because the EU aimed for an intra-Community market with the free movement of animals and their products, political pressure for a unified strategy at EU level increased. Despite reluctance among veterinarians and farmers to abandon the vaccination strategy, the EU decided to implement a FMD vaccination ban in 1992, which ended several decades of vaccination [20]. Cessation of the vaccination programme was only possible because internal (e.g. vaccine producing laboratories) and external (e.g. illegal trade of animals and animal products) sources of infection were considered to be of negligible risk. Also, high quality veterinary services were in place that enabled the transition to Stage I mitigation (pers. communication U. Kihm). A recurrence of FMD would trigger outbreak control measures as laid down in national contingency plans until the re-declaration of freedom from FMD.

In short, in the years preceding the start of the mitigation programmes by vaccination (1953), the feasibility of an intervention campaign was assessed and effective vaccines were developed (Stage II). After nearly 40 years of vaccination to reduce and ultimately eradicate disease (Stage III), a vaccination ban stipulated in 1992 enabled the transition to the sustainment stage. The mitigation programme for FMD in Switzerland that suffered its last FMD outbreak in 1980 illustrates prolonged Stage I sustainment after successful eradication.

##### A. Mitigation objective

Switzerland aims to sustain its FMD-free status as stipulated in the Swiss Animal Health Ordinance (SR 916.401).

##### B. Surveillance and intervention

Foot and mouth disease is notifiable, but there is no active surveillance programme in place. A national contingency plan is available that stipulates the response measures to be applied in the event of an outbreak. It lays down specific requirements regarding stamping out activities, hygiene, bio-security, cleaning and disinfection, and quarantine for the collection of milk and slaughtering in protection and surveillance zones. Furthermore, there are several mobile contingency teams available to cull and dispose of affected animals and clean and disinfect holdings. There are clear emergency disease reporting mechanisms in place, a transparent organisation and communication network, as well as an animal movement database. Responsibilities and collaboration on regional, national and international level are guaranteed.

##### C. Transition

In a first phase of a potential outbreak, Switzerland would strictly follow a stamping out policy supported by epidemiological simulation models. If the outbreak response measures failed and the disease spread widely, the situation would be re-assessed and a change in strategy towards a vaccination policy considered [21].

##### Summary

Switzerland is an example of a country that has been FMD free since successful eradication of the disease and aims at sustaining the free status (Stage I). This includes surveillance activities to ensure that an incursion can be recognised and outbreak response measure to avoid spread of the disease within the country in case it occurs.

## Conclusions

The proposed conceptual framework clarifies understanding of the key relationships between elements of mitigation and their technical characteristics, an essential precursor to economic analysis. The effectiveness of mitigation is usually measured in terms of prevalence or incidence reduction. But prevalence and incidence are not in themselves of economic interest. They matter because the lower are prevalence or incidence rates the greater the value, or benefits, obtained as outputs from resources committed to production.

Each of the three stages identified has been presented as a distinct phase in the sequential progression of a given pathogen from its first appearance through to its eventual control. In practice, mitigation for a defined pathogen in a target population (e.g. the poultry population of a country or region) can only be attributed to one stage at a time. However, a national or regional strategy for animal health, such as the EU's, will contain a mix of stages according to the status of the particular pathogens which are the foci of concern. Importantly, mitigation and its surveillance and intervention activities defined by technical considerations inevitably have implications for the data requirements of economic analysis.

Economic analysis of a national mitigation programme will need to take into account the costs and benefits of all essential components of the system. For example, an economic assessment of HPAI H5N1 mitigation in Vietnam would need to incorporate valuation of all economic consequences at national level due to the benefit losses from disease and the costs of its mitigation. These include the effects of morbidity and mortality in the human population, on-farm production losses due to mortality or culling of poultry, implications of movement restrictions for trade, consumption and resource use, and the financial costs of all surveillance and intervention activities (e.g. wage and salary payments, costs of test kits, sanitary measures, protective clothing, and vaccines). Upstream and downstream effects on businesses, for example breeders and slaughterhouses, as well as spill-over impacts on other sectors such as tourism also should be evaluated. Problems of food security in the short term would also have to be considered in a resource-poor economy with a large agricultural sector. The benefits would accrue from the avoidance of the negative economic consequences of loss of output and capacity to produce, the personal and wider social and economic implications of human illness or premature death, the risk from replication of such effects by the spread of infection to other countries, and the attendant resource expenditures made in the attempt to constrain these sources of lost well-being. For a national or international programme aimed at mitigating several different pathogens at once, identical principles apply.

For the initial sustainment stage to be a rational policy in economic terms, it must be based on an expectation that the future costs of failing to exclude a pathogen, or to maintain it at an acceptable level, will exceed recurrent sustainment costs. Such future costs are the present value of the sum of all future lost output value and all mitigation expenditure potentially incurred in Stages II and III as a result of failed sustainment now. Surveillance expenditures made now are to limit the need for future resource expenditures on intervention and surveillance by containing a pathogen's potential to cause future adverse output effects. In Stage I it is thus expected that surveillance is by far the dominant activity, and the main source of costs. Intuitively, recurrent surveillance expenditures of this kind are expected to be lower than the accumulated costs of failing to maintain a situation of exclusion or acceptability with respect to a given pathogen. However, the feasibility of implementing Stage I surveillance may differ considerably between developed and developing countries. For example, although wage rates for surveillance workers in labour-abundant developing countries are relatively low, skills may be lacking, increasing the risk of adverse consequences from Stage I failure. By contrast, the financial costs of Stage I surveillance may be high in developed economies, but their surveillance systems both technically and economically efficient.

But if Stage I fails, the inevitable result is a switch to a different approach to mitigation. Surveillance changes to focus on investigation, the better to inform resource expenditures when implementing intervention. In that sense, those additional resources committed to surveillance represent a cost of failed sustainment. If the purpose of the investigation stage is solely to inform Stage III implementation, potentially it can be attributed to implementation as a fixed cost necessarily incurred. From another perspective, if investigation adds to knowledge about the pathogen in such a way that the efficiency of mitigation is enhanced into the future, it becomes a long-term investment activity. Then, for given current investigation expenditures, lower current non-monetary benefits can be accepted because the total benefits that accrue to it do so not just now, but spread over an extended period of time into the more distant future.

In Stage III, the quantity of resources allocated to surveillance, and their specific technical characteristics, are primarily designed to inform the choice, timing, and scale of related interventions. They also document the progress of interventions in terms of their impact on prevalence or incidence reduction and, by implication, reduction in the economic value of output loss. Thus in contrast to Stage I sustainment where surveillance expenditures predominate, in Stage III intervention is expected to account for the greater proportion. Whether it does in fact is an empirical question.

A key economic consideration for all stages is to ascertain the least-cost combinations for surveillance and intervention, and the associated values of output losses thereby potentially avoided. By quantifying the relationship between surveillance, intervention, and corresponding prevalence or incidence rates, in principle it becomes possible to estimate prevalence or incidence rates that coincide with the economic optima for net benefits under different prices for outputs and resources expended. This is important, because however well-intentioned are target rates based exclusively on technical veterinary criteria, only by chance will they approximate to the best use of scarce resources or, in other words, the allocation that maximises people's economic well-being.

Finally, after successful intervention the mitigation objective may again be the sustained absence of disease. However, this is unlikely to be identical to the original Stage I mitigation. The difference is that now more information is available about pathogen effects, more having been learned as a result of investigation and implementation, and indeed perhaps why earlier sustainment failed in the first place. In that sense, the productivity of mitigation resources is enhanced by better knowledge, an unequivocal gain in economic efficiency.

### Practical applications

Conceptualising the technical relationship between elements of mitigation is the foundation on which to build the evaluation of mitigation as an economic process. It forms a basis for the development, testing and implementation of economic frameworks to assess the value of the three mitigation stages and is the precursor to economic analysis to identify strategies that maximise social net benefit.

Because diseases are part of biological systems, and therefore highly variable and complex, mitigation and thus surveillance activities need to be dynamic, adaptive and flexible over time, which is reflected in the approach presented. All surveillance programmes at one particular stage are likely to show similar characteristics, and so are expected to facilitate research and development of generic designs. For example, early warning systems in Stage I must be able to detect the incursion of a (re-)emerging or exotic pathogen quickly and must therefore be highly sensitive, which is likely to result in high costs. On the other hand, generally there is no need to act immediately on endemic diseases and more time can be spent to design surveillance programmes that provide fit-for-purpose data to prioritise and plan intervention activities. The situation is similar for documentation of disease freedom, where surveillance needs to detect the incidence or prevalence of disease for a defined level of confidence often stipulated in national or international legislation. In such situations of legislative constraint, the economic criterion would be to design and implement the most cost-effective programme consistent with achieving it.

The conceptual framework does not of itself provide any information about the most appropriate method of data collection, surveillance design, target pathogens or species. However, it supports decision-makers in identifying the stage of a mitigation process, and forms a sound conceptual basis to guide the collection of surveillance data. Based on the objective and stages of mitigation it reflects the real world setting within which decision makers need to operate, develop policy, and allocate their resources. The underlying assumption is that surveillance always informs mitigation. This helps to describe the goal of existing and putative mitigation targets clearly and outlines the need for surveillance to support that target. Hence, the understanding of the technical processes of mitigation may also be useful for algorithms for decision-making processes or comprehensive evaluation tools for government surveillance. Moreover, logical and sustained attention to such processes may facilitate the understanding of mitigation activities in other countries and the coordination of surveillance and intervention efforts at international level. This is of particular importance when dealing with the emergence and spread of highly infectious diseases, such as the recent outbreaks of influenza A virus subtype H1N1 and severe acute respiratory syndrome.

## Summary

The proposed conceptual framework set out above allows integration of the relationship between disease mitigation, a source of economic value, and mitigation resources, a source of economic cost, and thereby lays the foundation for applied economic analysis. Because the focus lies on the purpose of surveillance in relation to mitigation it is also expected to facilitate prioritisation and selection of the most appropriate surveillance and intervention activities. The formation of groups with similar characteristics may inform other research projects that aim at creating conceptual frameworks relating to surveillance, such as decision-making algorithms. Crucially, disease mitigation is both a technical problem and an economic problem, consideration of the latter dimension being relatively neglected. In a world of rapid population growth and increasing demand on scarce resources, allocating resources to surveillance and intervention in disease mitigation must aim for economic efficiency as well as technical efficiency.

## Appendix 1: Glossary

*- Surveillance *: The systematic ongoing collection, collation, and analysis of data related to animal health, objectively to inform decisions for the mitigation of public health hazards, and to demonstrate the absence of disease, infection or food-borne hazards (modified according to [8,22])

*- Surveillance system: *A method of surveillance that may involve one or more component activities that generates information on the health, disease or zoonosis status of animal populations [8].

*- Surveillance system component: *Has its self-contained surveillance protocol that focuses on a particular data source, such as serological bulk milk surveillance and surveillance of pathological lesions in the abattoir [10].

*- Surveillance approach: *Can be passive or active [23]. The selection of the surveillance approach is a key design decision because of its impact on bias and cost.

*- Surveillance design: *Describes activities and methods used for implementing, analysing and communicating surveillance system components, e.g. populations, sampling, diagnostics, case definition, and statistics.

*- Intervention: *The process of implementing measures directed at mitigation.

## Competing interests

The authors declare that they have no competing interests.

## Authors' contributions

BH, KH and KS developed the proposed conceptual framework. BH drafted the article and KH and KS read, critically revised and approved the final manuscript.

## Pre-publication history

The pre-publication history for this paper can be accessed here:

http://www.biomedcentral.com/1472-6963/11/225/prepub
